# Early-Onset Ramsay Hunt Syndrome After Pembrolizumab Administration in a Patient With Lung Adenocarcinoma

**DOI:** 10.7759/cureus.86935

**Published:** 2025-06-28

**Authors:** Satoshi Furuya, Shinnosuke Ikemura, Tsukasa Satoh, Masafumi Saiki, Kenzo Soejima

**Affiliations:** 1 Department of Respiratory Medicine, Graduate School of Medicine, University of Yamanashi, Chuo, JPN

**Keywords:** immune checkpoint inhibitor, immune-related adverse events, non-small lung cancer, pembrolizumab, ramsay hunt syndrome

## Abstract

Immune checkpoint inhibitors (ICIs) are associated with a broad spectrum of adverse effects referred to as immune-related adverse events (irAEs). Some infectious diseases can be precipitated by ICIs due to dysregulated inflammatory immune responses referred to as immune reconstitution inflammatory syndrome (IRIS), which were originally described in human immunodeficiency virus (HIV).

Here, we report a case of Ramsay Hunt syndrome (RHS) considered to have occurred as “non-HIV IRIS” after the administration of pembrolizumab. A 64-year-old man with stage IVb lung adenocarcinoma was treated with pembrolizumab as first-line chemotherapy. He presented with a headache in two days and right facial paralysis with some vesicles on the rash in the right external auditory canal six days after the administration. We diagnosed ICI-induced RHS and initiated valacyclovir and systemic corticosteroid therapy. The degree of facial paralysis was severe, but it recovered gradually.

Although ICI rarely causes RHS, clinicians should be aware of the possibility of RHS, as the prognosis of paralysis depends on early treatment.

## Introduction

Pembrolizumab is a human monoclonal antibody against programmed cell death 1 (PD-1), one of the immune checkpoint inhibitors (ICIs) [1]. PD-1 is expressed on the surface of T cells and inactivates T cells by binding its ligands. Inactivation of T cells prevents immune responses from attacking healthy cells, including cancer cells. Pembrolizumab can activate T cells by blocking the interaction of PD-1 and its ligands, and attack cancer cells and healthy cells. Pembrolizumab is indicated for the treatment of unresectable advanced non-small cell carcinoma by suppressing cancer cells [2] and is associated with various adverse reactions called immune-related adverse events (irAEs) by attacking healthy cells [3]. ICIs also cause infection and reactivation of viruses and bacteria [4].

Varicella-zoster virus (VZV) is one of the herpes viruses that causes chickenpox with a primary infection and establishes latency in the ganglion. Reactivation of latent VZV infection is called zoster, and reactivation in the geniculate ganglion is called Ramsay Hunt syndrome (RHS) [5]. RHS can lead to several symptoms, including unilateral facial nerve paralysis, hearing loss, and vesicular rash in the external ear canal. There are only a few reports on ICI-induced RHS [6]. In this report, we present a case of RHS occurring after the administration of pembrolizumab.

## Case presentation

In October 2021, a 64-year-old man, a past smoker with a Brinkman index of 860 and a history of postoperative angina and mitral regurgitation, was admitted to our hospital after a mass shadow was detected in the right middle lung field. His symptoms were a cough and right chest pain. A bronchoscopic examination confirmed the diagnosis of lung adenocarcinoma. Further evaluation with computed tomography (CT) and positron emission tomography (PET) scans identified multiple lung, mediastinal lymph node, bone, and pleural metastasis, leading to a final staging of cT2bN3M1c (stage IVB). The tumor cells had no druggable gene alterations but exhibited programmed death-ligand 1 (PD-L1) expression of more than 50%. He had no other immunosuppressive diseases or agents without cancer.

**Figure 1:**
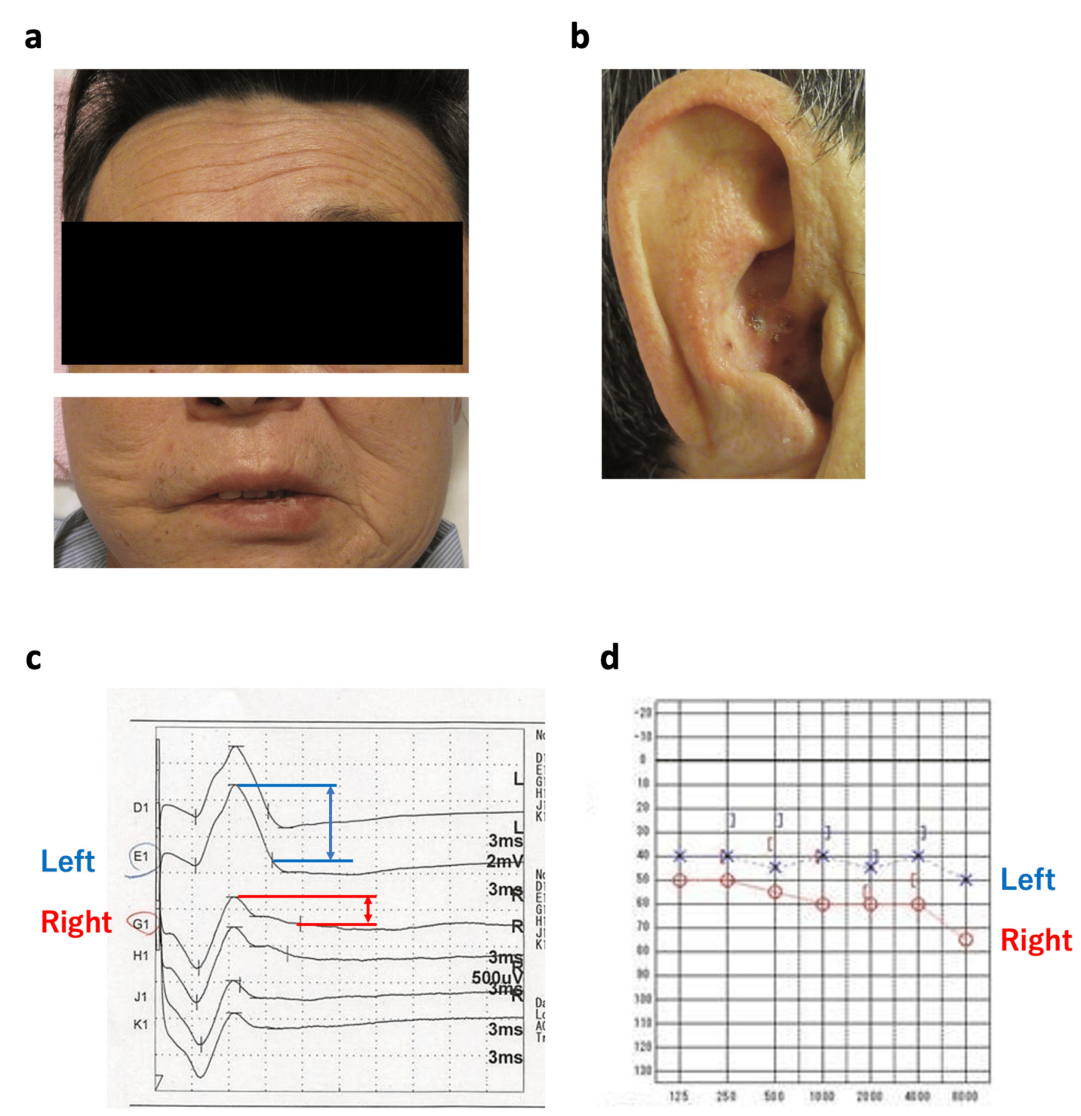
Facial nerve paralysis and sensory hearing loss A: On physical examination, weakness of his right face is seen: eyebrow sagging, disappearance of the nasolabial fold, and drooping at the corner of the mouth. B: The grade of his right facial paralysis was severe. In his right external ear canal, vesicles on rash are seen. C: Right facial nerve disorder was shown with evoked electromyogram. D: Audibility test showed right sensorineural hearing loss. Neurological disorders are seen in the right facial nerve and auditory nerve with zoster.

He was treated with pembrolizumab as first-line therapy. He presented with a headache in two days and right facial paralysis with some vesicles on rash in the right external auditory canal six days after the administration (Figure 1A, 1B). The degree of right facial paralysis was assessed as severe, with a score of 8/40 on the Yanagihara scoring system [7]. Laboratory tests showed elevated levels of serum C-reactive protein (0.39 mg/dL) (normal range (NR): <0.12 mg/dL) and varicella-zoster virus (VZV) IgM (7.02) (NR: <0.8) and IgG (124) (NR: <2.0) (Table 1). The right facial nerve disorder was confirmed through an evoked electromyogram (Figure 1C). The audibility test showed right sensorineural hearing loss (Figure 1D). Magnetic resonance imaging (MRI) of the head showed no intracranial lesions. Based on these findings, we diagnosed RHS as a disorder of the right facial nerve and auditory nerve caused by reactivation of VZV.

**Table 1:** Laboratory findings at the time of diagnosis of Ramsay Hunt syndrome VZV: varicella-zoster virus, GGT: gamma-glutamyl transferase

Laboratory findings	Patient value	Normal range
Albumin (g/dL)	3	4.1-5.1
Total bilirubin (mg/dL)	1	0.4-1.5
Alkaline phosphatase (U/L)	89	38-113
GGT (U/L)	27	<75
Lactate dehydrogenase (U/L)	220	124-222
Aspartate transaminase (U/L)	14	5-37
Alanine transaminase (U/L)	10	6-43
Blood urea nitrogen (mg/dL)	18	9-21
Creatinine (mg/dL)	0.61	0.6-1.0
C-reactive protein (mg/dL)	0.39	<0.12
VZV IgM	7.02	<0.8
VZV IgG	124	<2.0

**Figure 2:**
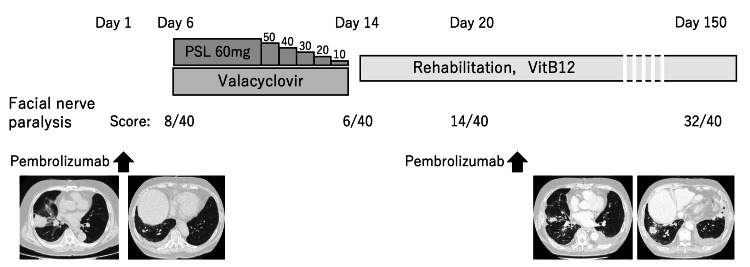
Clinical time course after the administration of pembrolizumab With the onset of RHS, prednisolone and valacyclovir were administered. Right facial nerve paralysis was severe, but it recovered gradually to mild paralysis. The scoring system used to assess the degree of facial paralysis is the Yanagihara scoring system (-8: severe, 10-18: moderate, 20-: mild). Chest imaging at baseline and after the second administration of pembrolizumab revealed that the lung adenocarcinoma progressed, except for the primary nodule, despite the administration of pembrolizumab. RHS: Ramsay Hunt syndrome, PSL: prednisolone

Corticosteroids and valacyclovir were administered against RHS from day 6 to day 14 after pembrolizumab initiation. The corticosteroid dose was tapered from 1 mg/kg to discontinuation over eight days. Although his headaches and vesicular rash resolved rapidly, dysfunction of the right facial and auditory nerves persisted. Following rehabilitation, his facial paralysis gradually improved, reaching a mild level with a score of 32/40 on the Yanagihara scoring system (Figure 2).

After the recovery of vesicles, pembrolizumab was restarted on day 22. There was no recurrence or worsening of RHS and no other irAEs. However, the lung cancer progressed, except for the primary nodule (Figure 2). Talc pleurodesis was performed for increased pleural effusion, and the systemic treatment was switched to chemotherapy with carboplatin and pemetrexed on day 59.

## Discussion

PD-L1 is expressed on tumor cells and is thought to negatively regulate the immune response by binding to PD-1 expressed on activated lymphocytes, resulting in the attenuation of anti-tumor immunity [1]. However, PD-L1 is also widely expressed on other cells, including immune and non-immune cells, and its expression is known to be upregulated during inflammation, such as viral infections.

Although immune checkpoint inhibitors (ICIs) activate T-cell-mediated immunity by blocking the PD-1/PD-L1 pathway and provide significant clinical benefits, they also result in immune-related adverse events (irAEs), which can include infectious diseases. However, ICIs have been reported to directly contribute to the development or worsening of certain infectious diseases through a hypersensitivity reaction akin to immune reconstitution inflammatory syndrome (IRIS) in individuals with human immunodeficiency virus (HIV), commonly referred to as “non-HIV IRIS” [8].

RHS is usually defined as a combination of facial paralysis, sensorineural hearing loss, and vesicles in the external auditory canal [5]. ICI-induced RHS has been reported in only a few case studies [6]. RHS is caused by the reactivation of latent VZV infection in the geniculate ganglion. Although immunosuppression can cause RHS in cancer patients, recent reports highlighted the correlation between VZV reactivation and ICI. It was found that the PD-1 antibody leads to a zoster at an equal rate to cytotoxic chemotherapy [9]. Additionally, a retrospective study disclosed that the PD-1/PD-L1 antibody may be an independent risk factor for zoster [10], although immunosuppressive drugs used against irAEs were also linked with zoster [11]. It is difficult to distinguish zosters from non-HIV IRIS because the timing of non-HIV IRIS varies widely from case to case [8,10].

**Figure 3:**
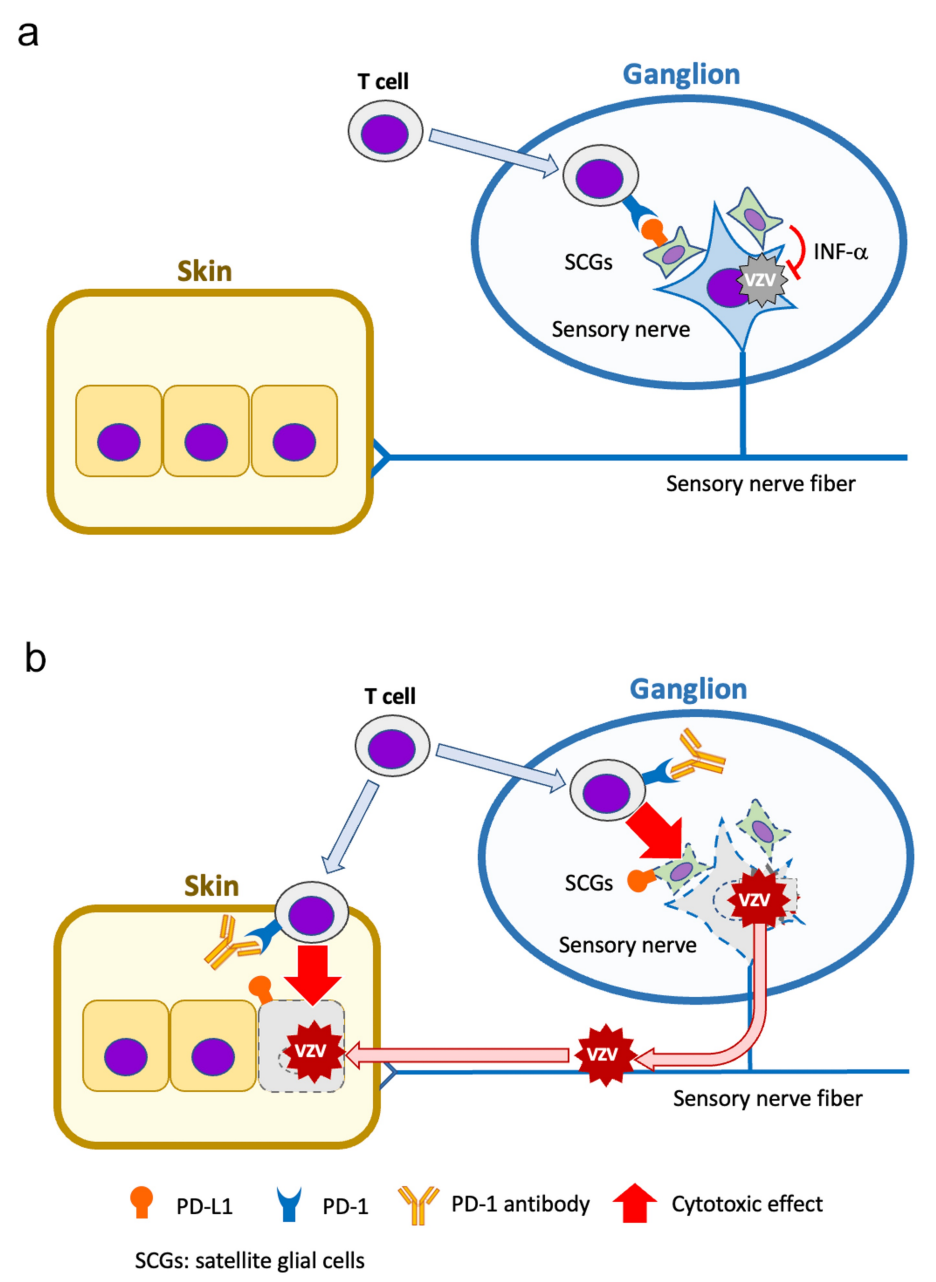
Schemas of VZV reactivation with the administration of PD-1 antibody A: SGCs suppress VZV reactivation via IFN-α production while maintaining latent infection and ganglionic protection through PD-1/PD-L1-mediated inhibition of T-cell activation. B: PD-1 antibody induces T-cell activation, leading to attacks on SGCs and sensory nerve cells, which results in ganglionic damage and VZV reactivation. Reactivated VZV is presumed to travel along sensory nerve axons to innervated skin epithelial cells. SGCs: satellite glial cells, VZV: varicella-zoster virus, IFN-α: interferon alpha, PD-1: programmed cell death 1, PD-L1: programmed death-ligand 1 This figure image was created by the authors.

The interaction between PD-1 on T cells infiltrating the ganglion and PD-L1 on satellite glial cells (SGCs) infected by VZV inhibits T-cell activation and protects neural cells in the ganglion [12]. The neural cells themselves lack the ability to produce interferon alpha (IFN-α), which controls the spread of VZV. However, SGCs are suggested to have the capacity to produce IFN-α and may play a role in regulating viral activation [13]. Specifically, SGCs surrounding the sensory nerve may suppress VZV reactivation through IFN-α production. Simultaneously, by inhibiting T-cell activation via PD-1 and PD-L1 interactions, SGCs are thought to maintain the latent VZV infection and the protective state of the ganglion (Figure 3A). PD-1/PD-L1 antibodies, however, induce T-cell activation, leading to attacks on SGCs and sensory nerve cells, which results in ganglionic damage and VZV reactivation. Reactivated VZV is then presumed to travel along sensory nerve axons to innervated skin epithelial cells (Figure 3B). Additionally, PD-L1 expression in VZV-infected epithelial cells has been reported to inhibit PD-1-expressing T-cell infiltration [14,15]. However, PD-1/PD-L1 antibody administration may trigger severe inflammation, leading to the formation of vesicles in the external auditory canal.

Contrary to this speculation, RHS did not recur following rechallenge with pembrolizumab. This may be due to the valacyclovir treatment suppressing the VZV sufficiently to control the RHS. Further research is needed to validate this speculation.

On the other hand, in terms of treatment, the skin symptom resolved quickly with the use of valacyclovir and steroids. However, the facial nerve paralysis did not fully recover, likely due to damage to the ganglion, and required long-term rehabilitation for improvement. Vaccination is widely used for the prevention of VZV reactivation. There are insufficient reports of VZV reactivation by ICIs to ascertain whether vaccination is advisable before treating ICIs [16]. Nonetheless, vaccination ought to be considered as RHS has the potential to cause constant facial paralysis.

## Conclusions

In summary, we have reported a rare case of RHS associated with lung adenocarcinoma treated with pembrolizumab. Latent VZV can be precipitated by ICIs, and physicians should be aware of RHS during immunotherapy, as it has the potential to cause persistent facial paralysis. Early diagnosis and treatment are effective in improving neurological prognosis, and vaccination is warranted for cancer patients before treatment including ICIs.
